# Primary omental torsion in children: case report

**DOI:** 10.11604/pamj.2013.14.57.1321

**Published:** 2013-02-11

**Authors:** Khattala Khalid, Tenorkorang Snr Somuah, Elmadi Aziz, Rami Mohamed, Bouabdallah Youssef

**Affiliations:** 1 Department of paediatric surgery, University Hospital Hassan II, Fez, Morocco

**Keywords:** omental torsion, childhood, surgery, emergency

## Abstract

Primary omental torsion is a rare cause of acute abdominal pain, we report a case of 10-year-old boy admitted with crampy abdominal pain, routine laboratory tests and plain abdominal radiography was normal, the patient underwent surgical exploration with the initial diagnosis of appendicitis, primary omental torsion was confirmed and the omentum was untwisted, with good postoperative evolution.

## Introduction

Primary omental torsion is a rare cause of acute abdominal pain. It clinical features may closely mimic that of acute appendicitis (acute onset of pain in the right lower abdominal quadrant). The Authors, after reporting one case recently observed and discuss it aetiopathogenetic aspects, diagnostic challenges and finally suggest the right surgical approach.

## Patient and observation

A 10-year-old boy was admitted to the Department of Paediatric Surgery of hospital university Hassan II with crampy abdominal pain which had lasted for a 5days. He also had nausea and vomiting in the 2 previous days associated with fever. The physical examination only showed tenderness and muscle guarding in the right lower quadrant of the abdomen. His Weight was at the 25th percentiles. The patient did not look ill as expected, although the duration of symptoms was relatively lengthy.

**Figure 1:**
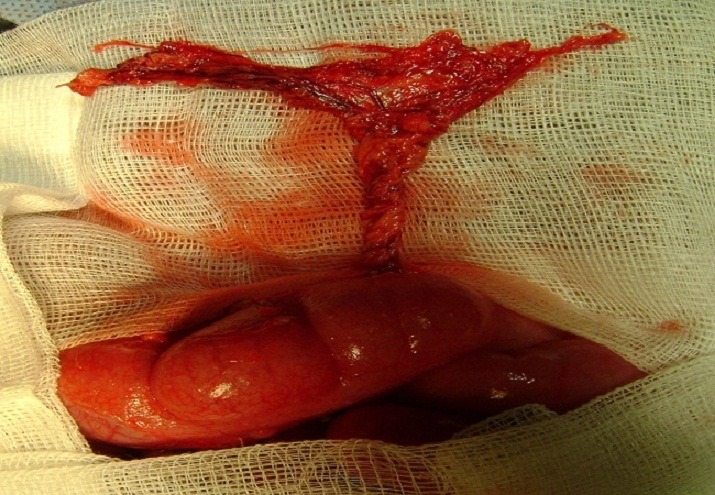
The omentum twisted 720 clockwise

Routine laboratory tests, including full blood count, and plain abdominal radiography, yielded normal results. After initial resuscitation, the patient underwent surgical exploration through a transverse incision in the right lower abdominal quadrant, with the initial diagnosis of appendicitis, 10 ml of clear serosangineous fluid was aspirated from the peritoneal cavity. A thick, firm, knobby mass was then encountered in the right lower abdominal cavity. This mass was found to be an edematous omentum. The omentum was twisted 720 clockwise, without any point of distal fixation (Figure 1). The omentum was untwisted. Through exploration of the cavity found a normal appendix without any additional findings. No growth of microorganisms was encountered from the peritoneal culture. There were no complications after the operation, and the patient was discharged on the fourth postoperative day.

## Discussion

Primary omental torsion (POT), known since 1899 [1], is a disease that occurs in the fourth and fifth decades of life. Although it is a rare cause of acute abdominal pain in children [1–3], the paediatric surgeon should be aware of this diagnostic possibility.

The etiology of POT is obscure, some predisposing factors, such as anatomic variations including the malformations of omental pedicle, tonguelike projections along the free edge of the omentum, bifid omentum, accessory omentum, and omental fat associated with obesity, are suggested in the etiology. Others include: trauma, overeating, overexertion, sudden change in position, coughing and straining, Obesity is an important risk factor for POT in children [3–6] and the presence of inflammatory focus have been suggested to have a role in the etiopathogenesis [1, 5]. However, none of the suggested anatomic variations or factors was encountered in our patient. In the paediatric age group, POT is an uncommon cause of acute abdominal pain [6], which is diagnosed during surgical exploration. Over a two-year period, 54 laparoscopic explorations for acute abdominal pain in children have been performed, in two cases primary torsion of the greater omentum was the underlying cause, the authors believe that primary omental torsion was underestimated, because many cases were not recognised, even during laparotomy for appendicitis [6, 7]. This is usually sudden in onset, without radiation. Localization of the pain depends on the portion and size of the omentum undergoing rotation [1]. Regardless of the original localization of the pain, it invariably becomes localized to the right side of the abdomen. Anorexia, nausea, vomiting, and diarrhea may occur. In addition to the presence of tenderness over the involved segment of the omentum, usually in the lower quadrants, muscle guarding also may be encountered. Although this clinical picture closely mimicks that of acute appendicitis [1, 6], the patients with POT are not as ill as expected according to the duration of the symptoms [5]. Sometimes ultrasonography and computed tomography can establish the diagnosis safely and allow conservative management [6, 8]. The lesions are hyperechoic or of mixed attenuation. Aoun et al [1] has reported a case of omental torsion which was preoperatively diagnosed by CT. In the present case, ultrasonographic findings were evaluated as normal. Presence of a normal appendix at surgery should raise the suspicion of omental torsion when facing primary omental torsion, Oğuzkurt et al [1] considered the presence of sterile, serosanguinous fluid within the peritoneal cavity a universal finding. This was observed in our patient and the fluid culture was sterile. In the other ways, laparoscopy is a great help for the diagnosis and the treatment [5, 8].

Concerning the treatment of omental torsion, resection of the involved segment of the omentum has traditionally been the treatment of choice [1]. However, a few reported cases of omental torsion that were successfully treated conservatively have been reported us in our case [1, 9], especially in patients without associated complications. This may suggest that surgical treatment should be limited to the patients with complications. However, in cases where conservative treatments are not effective, as in our case, surgical treatments should be considered.

## Conclusion

Omental torsion is a rare cause of acute abdominal pain. The diagnosis is usually not established before surgery. Resection of the involved segment of the omentum has traditionally been the treatment of choice.
